# C-Reactive Protein Is a Potential Prognostic Marker in Patient with Advanced or Metastatic Urothelial Carcinoma Treated with Enfortumab Vedotin: A Multi-Center Retrospective Study

**DOI:** 10.3390/cancers16091725

**Published:** 2024-04-28

**Authors:** Toshiharu Morikawa, Taku Naiki, Yosuke Sugiyama, Aya Naiki-Ito, Takashi Nagai, Toshiki Etani, Keitaro Iida, Teruki Isobe, Yusuke Noda, Nobuhiko Shimizu, Maria Aoki, Masakazu Gonda, Rika Banno, Hiroki Kubota, Ryosuke Ando, Yukihiro Umemoto, Noriyasu Kawai, Takahiro Yasui

**Affiliations:** 1 Department of Nephro-Urology, Graduate School of Medical Sciences, Nagoya City University, Nagoya 467-8601, Japan; t-mrkw@med.nagoya-cu.ac.jp (T.M.); tkshng73@med.nagoya-cu.ac.jp (T.N.); uroetani@med.nagoya-cu.ac.jp (T.E.); k-iida@med.nagoya-cu.ac.jp (K.I.); isobe-11@med.nagoya-cu.ac.jp (T.I.); yusukenoda0401@gmail.com (Y.N.); shdasif1@gmail.com (N.S.); hd.maria@med.nagoya-cu.ac.jp (M.A.); mgonda@med.nagoya-cu.ac.jp (M.G.); ryo@med.nagoya-cu.ac.jp (R.A.); uro-ume@med.nagoya-cu.ac.jp (Y.U.); n-kawai@med.nagoya-cu.ac.jp (N.K.); yasui@med.nagoya-cu.ac.jp (T.Y.); 2 Department of Urology, Nagoya City University West Medical Center, Nagoya 467-8601, Japan; 3 Department of Pharmacy, Nagoya City University Hospital, Nagoya 467-8601, Japan; phsugi@sunprom.med.nagoya-cu.ac.jp; 4 Department of Experimental Pathology and Tumor Biology, Graduate School of Medical Sciences, Nagoya City University, Nagoya 467-8601, Japan; ayaito@med.nagoya-cu.ac.jp; 5 Department of Urology, Anjo Kosei Hospital, Anjo 446-8602, Japan; 6 Department of Urology, Konan Hospital, Konan 483-8704, Japan; rikabanno@gmail.com; 7 Department of Urology, Kainan Hospital, Yatomi 498-8502, Japan; 479659@kainan.jaaikosei.or.jp

**Keywords:** enfortumab vedotin, geriatric nutritional risk index, advanced or metastatic urothelial carcinoma, late line treatment

## Abstract

**Simple Summary:**

We aimed to investigate potential prognostic markers for enfortumab vedotin therapy in Asian patients with advanced or metastatic urothelial carcinoma. We retrospectively enrolled 61 Japanese patients treated with enfortumab vedotin therapy and analyzed overall survival, adverse events, and potential prognostic markers. Enrolled patients (38 men, 23 women; median age 74 [IQR: 68–79] years) had bladder cancer (26 patients) or upper-tract urothelial carcinoma (35 patients). Our study provides real-world data showing that enfortumab vedotin prolonged survival in Asian patients similar to the EV-301 trial. Additionally, the C-reactive protein level might be considered a prognostic marker of enfortumab vedotin therapy in such patients.

**Abstract:**

Background: In the EV-301 trial, enfortumab vedotin prolonged survival in patients with locally advanced or metastatic urothelial carcinoma previously treated with platinum-based therapy and programmed cell death 1/programmed death-ligand 1 inhibitor. However, real-world Asian data are limited, and potential prognostic markers are non-existent. We aimed to investigate potential prognostic markers for enfortumab vedotin therapy in Asian patients. Methods: We retrospectively enrolled 61 Japanese patients treated with enfortumab vedotin therapy at our hospital and affiliated hospitals between January 2019 and September 2023. Results: Enrolled patients (38 men, 23 women; median age 74 [IQR: 68–79] years) had bladder cancer (26 patients) or upper-tract urothelial carcinoma (35 patients). Fifty-four patients reported adverse events (grade >3 in 12). Skin disorders, pruritus, and neuropathy were common adverse effects. The median overall survival was 17.1 months (95% confidence interval: 10.0–not applicable). In multivariate analysis, the C-reactive protein level was an independent marker predicting favorable overall survival with enfortumab vedotin. Patient characteristics did not differ between C-reactive protein-high and -low groups. Conclusions: Our study provides real-world data showing that enfortumab vedotin prolonged survival in Asian patients similar to the EV-301 trial. Additionally, the C-reactive protein level might be considered a prognostic marker of enfortumab vedotin therapy in such patients.

## 1. Introduction

Urothelial carcinoma (UC) is a frequently occurring cancer in genitourinary organs that results in considerable deaths worldwide [1]. Immune checkpoint inhibitors (ICIs) that target programmed cell death 1/programmed death-ligand 1 (PD-L1), including pembrolizumab, nivolumab, and atezolizumab, are used for locally advanced or metastatic UC (la/mUC). However, treatment strategies for patients with la/mUC remain unsatisfactory; most patients invariably progress and require other systemic therapy for disease control [2,3,4,5]. Enfortumab vedotin (EV) is an antibody conjugated drug. It is made up of a monoclonal antibody that targets nectin-4 and is conjugated to the microtubule-disrupting agent, monomethyl auristatin E. Enfortumab vedotin is available after the failure of platinum-based chemotherapy and ICI in patients with la/mUC on the basis of the excellent findings of the EV-301 trial [6,7]. Based on just over two years of recent follow-up results, the risk of death was reduced by 30% with EV versus chemotherapy (hazard ratio [HR]: 0.70; 95% confidence interval [CI]: 0.58–0.85, *p* = 0.00015]. Progression-free survival (PFS) also improved with EV (HR: 0.63; 95% CI: 0.53–0.76, *p* < 0.00001) [8]. However, unlike for patients in a clinical study setting, in clinical practice, the backgrounds of patients are distinct and variable. Additionally, the analysis of potential biomarkers to predict eligibility for receiving EV treatment was limited.

The biomarkers ICIs and EV have been determined in many investigations for the prognosis of patients with la/mUC treated with platinum-based chemotherapy [9]. One such marker is the immune-nutritional status, which is related to a patient’s prognosis and health during treatment. Of the identified biomarkers, we studied the Geriatric Nutritional Risk Index (GNRI), which evaluates nutrition and is based on the ratio of actual to ideal body weight plus serum albumin level [10,11,12]. Patients with la/mUC and showing a high GNRI at the beginning of initial treatment had a superior prognosis to that of patients showing a low GNRI. Additionally, when the GNRI is high, this may predict a good prognosis in those patients with la/mUC treated with ICIs. Moreover, several studies showed that the serum C-Reactive Protein (CRP), a marker of inflammation, was a prognostic marker in the systemic therapy including EV for la/mUC [13,14,15,16,17,18,19]. However, there was no evidence as to whether the GNRI or CRP was a better prognostic marker of EV therapy.

Therefore, in a retrospective multi-center study, we assessed the safety and efficacy of EV treatment based on data from patients with la/mUC. Furthermore, in such patients, we assessed the significance of immune-nutritional parameters, including the GNRI, in prognosis.

## 2. Materials and Methods

### 2.1. Patient Enrollment

**Figure 1:**
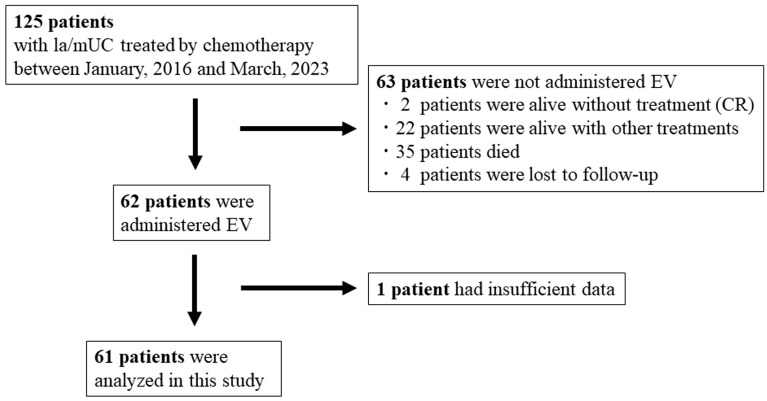
Patients’ enrollment in the total cohort (*n* = 61). CR: Complete response, EV: Enfortumab vedotin, la/mUC: locally advanced or metastatic urothelial carcinoma.

Patients treated for la/mUC found in their upper urinary tract or urinary bladder at Nagoya City University Hospital or seven associated institutions between January 2016 and March 2023 were enrolled in this study. A diagnosis was made after specimens were examined histologically. The selection criteria for this study were as follows: (1) an la/mUC diagnosis and a minimum of one cycle of first-line chemotherapy; (2) enhanced computed tomography (CT) was used to stage lesions, or plain CT was used if enhanced CT was not practical; (3) primary lesions that were biopsied or surgically removed; and (4) after switch maintenance treatment with avelumab, second-line treatment using chemotherapy, or pembrolizumab failed, patients received third- or later-line EV treatment. As shown in the flowchart in Figure 1, during this period, a total of 125 patients with la/mUC were treated with first-line chemotherapy. After excluding 64 patients with either no additional treatment, who were alive without treatment, who died without treatment, who were lost to follow-up, or had missing data, 61 patients remained who were included in this study. Data were collected and assessed retrospectively. Our retrospective study was approved by the Ethics Committee of Nagoya City University Hospital (Approval No. 60-18-0060). We used specimens that were derived from routine pathological samples collected in the past. Patients had the opportunity to opt out of this investigation. This study was conducted with respect to the Declaration of Helsinki (2013 Fortaleza revision).

### 2.2. Treatment

All patients received a dose of EV at 1.25 mg/kg on days 1, 8, and 15 of each 28-day cycle. When grade 3 adverse events (AEs) occurred, the dose was reduced to 1.0 mg/kg from the next cycle. The sizes of tumors were determined by CT and physical examinations were conducted on patients. Patient responses to treatments were assessed according to The Response Evaluation Criteria In Solid Tumors (RECIST), version 1.1 after two cycles of EV. The objective response rate (ORR) was determined from the ratio of the number of patients with a complete (CR) or partial (PR) response to the total number of patients who were treated. Treatment was stopped if disease progressed according to RECIST criteria or AEs developed that were not well tolerated. The PFS was defined as the time from the start of EV therapy to treatment progression. The period of time from the start of EV therapy until when a patient died due to mUC was the overall survival (OS). Adverse events were reported according to the National Cancer Institute Common Terminology Criteria for AEs, version 5.0. We collected information on primary cancer pathology, serum albumin levels, performance status, age, body mass index (BMI), peripheral blood counts, AEs, and gender.

### 2.3. EV-Treated Patients Were Evaluated Based on Clinical Characteristics, CRP, NLR, and GNRI as Prognostic Factors for EV Treatment

**Figure 2:**
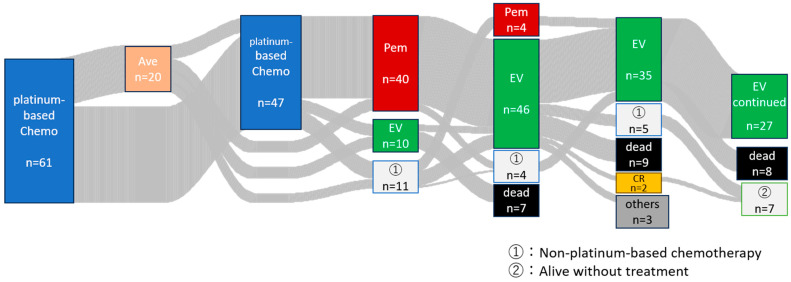
Treatment flow of patients in this cohort. Ave: Avelumab, Chemo: Chemotherapy, CR: Complete response, EV: Enfortumab vedotin, PEM: Pembrolizumab.

We retrospectively analyzed patients who received EV as a third- or later-line treatment. Figure 2 shows the treatment flow for 61 patients in total who underwent EV therapy. Values for the geriatric nutritional risk index were determined as follows: 14.89 × serum albumin level (g/L) + 41.7 × (actual body weight [kg]/ideal body weight [kg]). A ratio of actual to ideal body weight where the actual body weight > ideal body weight was given a value of one. Ideal body weight was equal to 22 × height (m^2^) [10]. The cut-off level of GNRI was classified by a receiver operating characteristics (ROC) curve targeted to an objective response (CR+PR). Blood samples were collected just before the EV administration. Levels of CRP were also classified by a cut-off level according to the ROC curve for peripheral blood markers. The neutrophil–lymphocyte ratio (NLR) was calculated based on the ratio of an absolute neutrophil count (/µL) to absolute lymphocyte count (/µL), and also classified by a cut-off level by ROC curve.

### 2.4. Statistics

**Figure 3:**
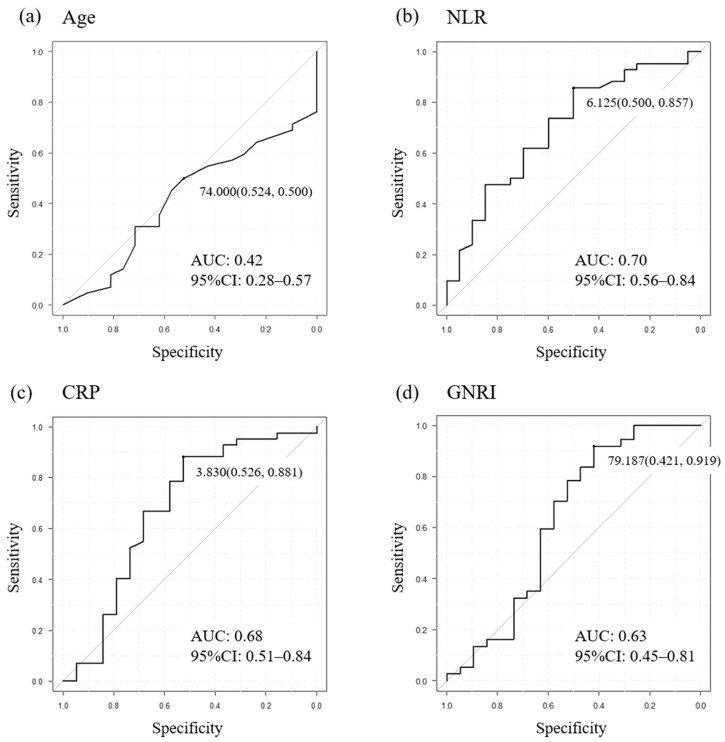
Setting cut-off values using ROC curve analysis of indicators in the total cohort targeting an objective response to EV. Age (**a**), NLR (**b**), CRP (**c**), and GNRI (**d**). AUC: Area under the curve, CI: Confidence interval, CRP: C-reactive protein, EV: Enfortumab vedotin, GNRI: Geriatric nutritional risk index, NLR: Neutrophil–lymphocyte ratio, ROC: Receiver operating characteristic.

A Fisher’s exact test or Mann–Whitney *U* test was used for differences in categorical parameters. Kaplan–Meier curves were used to determine cumulative rates of OS. A log-rank test was used to calculate significant differences. Cox proportional hazard regression analyses were used to calculate univariate and multivariate analyses; the variables showing *p* < 0.01 in the univariate Cox proportional hazard regression model were entered into the multivariate Cox regression covariates. A cut-off value for each prognostic factor was determined by ROC curve targeted to an objective response as outlined in Figure 3. By ROC analysis using Youden’s index, the cut-off value was calculated as age (≥74 vs. <74 years), CRP level (≥3.8 vs. <3.8 mg/dL), NLR (≥6.1 vs. <6.1), and GNRI status (≥80.0 vs. <80.0). For baseline parameters, age and gender were added as baseline prognostic factors as determined in previous investigations [10,11,12].

Statistical significance was considered *p* < 0.05. Two-tailed *p* values were used. Data were analyzed using an EZR statistical program from the Saitama Medical Center, Jichi Medical University (Saitama, Japan). This program is a graphical user interface for R (The R Foundation for Statistical Computing, Vienna, Austria) [20].

## 3. Results

### 3.1. Patient Charcteristics and Their Oncological Outcomes

**Figure 4:**
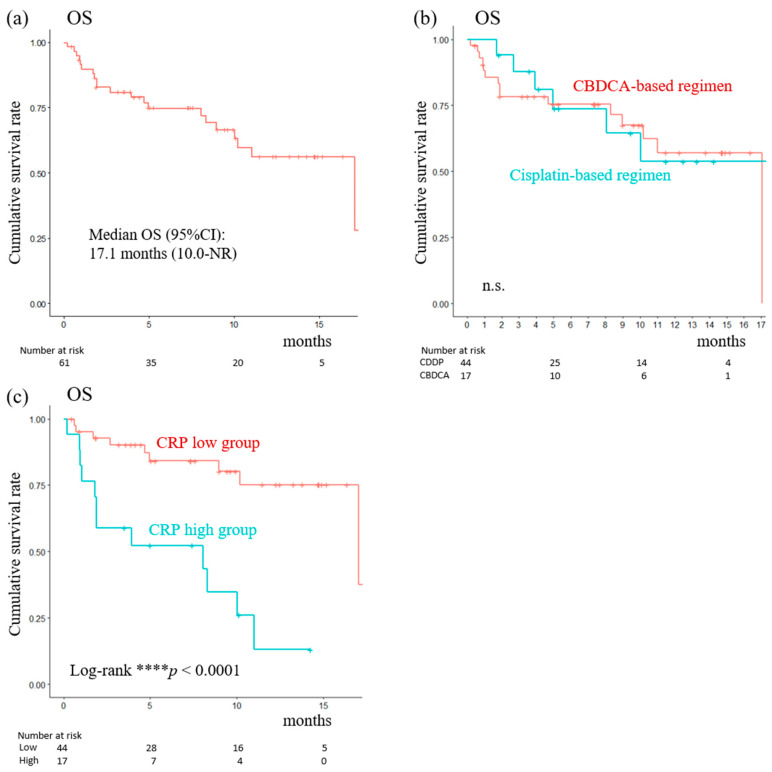
OS from the start of EV treatment in the total cohort (**a**) and between cisplatin-based and carboplatin (CBDCA)-based regimens as first-line treatment (**b**) of Kaplan–Meier curves analyzing indicators. OS between CRP-high and CRP-low groups from the start of EV treatment (**c**). CI: Confidence interval, CRP: C-reactive protein, EV: Enfortumab vedotin, NR: Not reached, n.s.: Not significant, OS: Overall survival, **** *p* < 0.0001 for CRP-high vs. CRP-low groups.

In this study, the total number of patients with la/mUC who were enrolled and who were treated with EV as a third- or later-line treatment was 61. Patients’ characteristics are noted in Table 1. As shown in Table 2, univariate and multivariate analyses revealed that the CRP level was the sole independent prognostic factor predicting better OS among baseline and serum-based parameters. Therefore, we divided the total cohort into CRP-high and CRP-low groups based on the cut-off value. Consequently, 44 patients were in the CRP-high group and 17 patients were in the CRP-low group. Table 3 shows patients’ characteristics in the two groups. The two groups did not show any statistical difference when starting first-line treatment for the following: age, distribution of gender, a first-line chemotherapy regimen, and primary and metastatic sites. The CRP-low group was better than the CRP-high group in terms of the median number of cycles and response to first-line chemotherapy. In addition, Eastern Cooperative Oncology Group performance status, inflammation, and nutritional status, including NLR and GNRI status, were superior in the CRP-low group. In the total cohort, the median OS from the initiation of EV treatment was 17.1 months (95% CI: 10.0–not reached [NR]; Figure 4a). In addition, the median OS in terms of the regimen of first-line chemotherapy was similar (Figure 4b). In addition, the median OS was significantly superior in the CRP-low (median 17.7 months, 95% CI: 17.1–NR) compared to the CRP-high (median 8.0 months, 95% CI: 1.1–11.0; Figure 4c) group.

### 3.2. Adverse Events

Table 4 outlines AEs associated with EV treatment between the two groups. With regard to hematological AEs, anemia was the most frequent. Of non-hematological AEs, skin disorders, pruritus, neuropathy, and dysgeusia were found to be the most frequent AEs. Compared with the CRP-high group, neuropathy and dysgeusia occurred significantly more frequently in the CRP-low group. Skin disorders, pruritus, and fatigue tended to be more frequent in the CRP-low compared to the CRP-high group. However, the incidences of grade 3–4 AEs were similar between the two groups. These AEs were controllable and treatment-related deaths were not noted. The timing of onset of each AE are listed in Table 5. Most AEs were recognized within three cycles of EV. However, some, neuropathy in particular, emerged after five or more cycles.

## 4. Discussion

In this retrospective study of mUC, third- or later-lines of EV treatment contributed to a greater than one-year OS with acceptable AE profiles. Since the findings of the EV-301 trial were published, clinical data relating to EV therapy for la/mUC have gradually been published in many countries [21]. For example, as a representative study in Western countries, a large multi-institutional retrospective study (Urothelial Cancer Network to Investigate Therapeutic Experiences [UNITE] study) enrolled 304 patients from 16 academic institutions [22]. In the UNITE study, the median age of patients was 70 years. The ORR was 52% for all patients. The median PFS and OS were 6.8 months (95% CI: 5.6–7.4) and 14.4 months (95% CI: 11.8–16.9), respectively. However, the PD-L1 status or tumor mutations were not found to be prognostic biomarkers. Only the existence of liver metastasis showed significantly worse OS compared to its absence (8.3 months [95% CI: 6.7–15.3] vs. 15.7 months [95% CI: 12.3–19.7], respectively). In Japan, as the largest retrospective real-world study, Fukuokaya et al. enrolled 103 evaluable patients who received EV treatment. The ORR was found to be 50.5%, the median PFS was 6.0 months (95% CI: 4.7–9.8), and the median OS was 14.5 months (95% CI: 12.4–NR) with acceptable AE profiles [23]. Such data highlighted the acceptable safety profile and oncological outcomes of EV therapy in patients with la/mUC. However, the biomarker analysis of EV treatment was very limited [24,25,26]. In the past, potential prognostic variables were identified that included a variety of inflammation and nutritional markers such as CRP, NLR, and the platelet–lymphocyte ratio [16,17,18,19]. In particular, concerning the value of the prognostic biomarker of CRP in the analysis, Klumper et al. highlighted that the CRP response predicts immunotherapy response and outcomes in mUC independently in the 154 patients with mUC in the multi-center observational study by [16]. Only recently, Inoue et al. described the prognostic value of CRP in patients treated with immunotherapies including pembrolizumab, avelumab, and EV by retrospective analysis. The novel aspect of this study was that three immune-nutritional-related factors and baseline parameters were analyzed; the CRP level was found to be the sole independent prognostic factor for better OS. In addition, the median OS of the CRP-low group was significantly better than that of the CRP-high group. This result differs from our previously reported study that high GNRI may be a better prognostic marker in patients with mUC treated with cisplatin-based chemotherapy or ICIs compared to CRP or NLR before previous treatment [11,12]. For this reason, in patients receiving third- or later-line treatment who could tolerate the cachexia status due to treatment with a long-term remedy and primary disease, the inflammation status may be superior in predicting the prognosis compared with the nutritional status [27]. The accumulation of further information on patients may lead to the identification of the best possible characteristics of sequential therapy, and therefore, an appropriate therapy for mUC. In this study, though the total number of AE profiles was acceptable, the incidence of non-hematological AEs, including neuropathy and dysgeusia, was found to be significantly higher in the CRP-low compared to the CRP-high group. One reason for this may be due to differences in the median cycles of EV treatment (five cycles for the CRP-low vs. three cycles for the CRP-high group). None of the patients interrupted a course of EV treatment due to AEs. In the CRP-high group, the cessation of EV treatment was due to the progression of primary disease, similar to the EV-301 trial. Taking care that neuropathy may emerge in every cycle, and that a dose reduction may be managed at an appropriate time, third- or later-line EV treatment is recommended, even in Asian patients.

This investigation had several limitations. First, as a retrospective analysis, a selection bias might have influenced the prognosis. Second, a small sample size was used. Our modest sample size resulted in a wide 95% confidence interval for the area under the ROC curve. A larger study would yield a more accurate estimation of the true area under the ROC curve. Third, a relatively short follow-up period was used. Despite these shortcomings, CRP may be viewed as an effective practical prognostic biomarker and can predict survival outcomes in patients with la/mUC receiving EV treatment.

## 5. Conclusions

In summary, we found that third- or later-line EV treatment prolonged OS in patients with la/mUC, showing a tolerable safety profile; this was also found by others. Additionally, CRP was useful as a biomarker to predict an objective response and better prognosis of EV treatment.

## Figures and Tables

**Table 1:** Patients’ characteristics in the total cohort.

Characteristics		Overall (*n* = 61)
Median age, years (IQR)		74 (68–79)
Median BMI, kg/m^2^ (IQR)		22.0 (19.9–25.0)
Gender, *n* (%)	Male	38 (62.3)
	Female	23 (37.7)
Primary tumor, *n* (%)	Bladder	26 (42.6)
	Upper tract	35 (57.4)
Regimen of first-line chemotherapy, *n* (%)	GC	44 (72.1)
	GCarbo	17 (27.9)
Median number of cycles in first-line chemotherapy (IQR)		4 (3–6)
Response to first-line chemotherapy, *n* (%)	CR	0 (0)
	PR	25 (41.0)
	SD	24 (39.3)
	PD	12 (19.7)
Previous ICI treatments	Avelumab	11 (20.0)
	Pembrolizumab	35 (63.6)
	Both	9 (16.4)
ECOG-PS at the start of EV treatment, *n* (%)	0	11 (18.0)
	1	31 (50.8)
	Above 2	19 (31.1)
Median cycles of EV treatments, cycles, *n* (IQR)		4 (2–8)
Metastatic lesions at the start of EV treatment, *n* (%)	Lymph node	35 (57.4)
	Lung	24 (39.3)
	Liver	7 (11.5)
	Bone	25 (41.0)
Serum markers at the start of EV treatment, *n* (%)	LDH	196 (174–251)
	NLR	3.07 (1.81–6.50)
	GNRI	88.7 (82.0–98.5)

BMI: Body mass index, CR: Complete response, ECOG-PS: Eastern Cooperative Oncology Group performance status, EV: Enfortumab vedotin, GC: Gemcitabine and cisplatin, GCarbo: Gemcitabine and carboplatin, GNRI: Geriatric nutritional risk index, ICI: Immune checkpoint inhibitors, LDH: Lactate dehydrogenase, NLR: Neutrophil–lymphocyte ratio, PD: Progressive disease, PR: Partial response, SD: Stable disease.

**Table 2:** Univariate and multivariate analyses of baseline and serum-based parameters predict the better OS in the 61 patients in the total cohort.

Parameters	Survival Event in Univariate	Univariate			Multivariate		
		HR	95% CI	*p* Value	HR	95% CI	*p* Value
Age, ≥74 vs. <74 years	13/30	0.57	0.23–1.38	0.21	-	-	-
Gender, male vs. female	7/23	0.71	0.28–1.82	0.48	-	-	-
NLR status, ≥6.1 vs. <6.1	10/43	3.61	1.49–8.74	0.004 **	1.52	0.48–4.75	0.47
CRP level, ≥3.8 vs. <3.8	9/44	5.32	2.14–13.16	0.0003 ***	4.09	1.28–13.1	0.018 *
GNRI status, ≥80 vs. <80	6/12	0.36	0.13–0.98	0.04 *	-	-	-

CI: Confidence interval, CRP: C-reactive protein, GNRI: Geriatric nutritional risk index, HR: Hazard ratio, NLR: Neutrophil–lymphocyte ratio. * *p* < 0.05, ** *p* < 0.01, *** *p* < 0.001, statistically significant.

**Table 3:** Comparison of patients’ characteristics in CRP-low and CRP-high groups.

Characteristics		CRP-Low Group (*n* = 44)	CRP-High Group (*n* = 17)	*p* Value
Median age, years (IQR)		74 (67–78)	74 (68–79)	0.61
Median BMI, kg/m^2^ (IQR)		22.1 (20.0–25.3)	21.4 (19.6–24.6)	0.62
Gender, *n* (%)	Male	27 (61.4)	11 (64.7)	1.0
	Female	17 (38.6)	6 (35.3)	
Primary tumor, *n* (%)	Bladder	18 (40.9)	8 (47.1)	0.76
	Upper tract	26 (59.1)	9 (52.9)	
Regimen of first-line chemotherapy, *n* (%)	GC	31 (70.5)	13 (76.5)	0.76
	GCarbo	13 (29.5)	4 (26.5)	
Median number of cycles in first-line chemotherapy, *n* (IQR)		4 (3–6)	3 (1–4)	<0.05 *
Response to first-line chemotherapy, *n* (%)	CR	0 (0)	0 (0)	<0.05 *
	PR	22 (50.0)	3 (17.6)	
	SD	16 (36.4)	8 (47.1)	
	PD	6 (13.6)	6 (35.3)	
Previous ICI treatments, *n* (%)	Avelumab	8 (20.0)	3 (20.0)	1.0
	Pembrolizumab	25 (62.5)	10 (66.7)	
	Both	7 (17.5)	2 (13.3)	
ECOGPS at the start of EV treatment, *n* (%)	0	11 (25.0)	0 (0)	<0.05 *
	1	23 (52.3)	8 (47.1)	
	2	10 (22.7)	9 (52.9)	
Median cycles of EV treatments, cycles (IQR)		5 (3–8)	3 (1–5)	0.052
Metastatic lesions at the start of EV treatment, *n* (%)	Lymph node	26 (59.1)	9 (52.9)	0.78
	Lung	18 (40.9)	6 (35.3)	0.78
	Liver	4 (9.1)	3 (17.6)	0.39
	Bone	16 (36.4)	9 (52.9)	0.26
Serum markers at the start of EV treatment, *n* (%)	LDH	202 (132–2160)	187 (130–4386)	0.89
	NLR	2.74 (0.86–40.33)	9.38 (0.64–55.33)	<0.001 ***
	GNRI	91.9 (66.8–124.2)	81.1 (55.7–96.4)	<0.001 ***

BMI: Body mass index, CR: Complete response, CRP: C-reactive protein, ECOG-PS: Eastern Cooperative Oncology Group performance status, EV: Enfortumab vedotin, GC: Gemcitabine and cisplatin, GCarbo: Gemcitabine and carboplatin, GNRI: Geriatric nutritional risk index, ICI: Immune checkpoint inhibitors, LDH: Lactate dehydrogenase, NLR: Neutrophil–lymphocyte ratio, PD: Progressive disease, PR: Partial response, SD: Stable disease, * *p* < 0.05, *** *p* < 0.001, statistically significant.

**Table 4:** Comparison of number of incidences of adverse events in retrospective cohorts between CRP-low and CRP-high groups. For hematological AEs, the incidence of grade 3–4 AEs was statistically compared between the two groups. For non-hematological AEs, the total incidences of AEs were statistically compared between the two groups.

Adverse Events		No. of Cycles of EV at Onset of Each AE				
		1	2	3	4	5 or More (Emerged Cycles)
Hematological	Neutropenia	4				
	Anemia	4	3	2		
	Thrombocytopenia					
Non-hematological	Skin disorders	24	9	2	2	
	Pruritus	15	4	1	2	
	Neuropathy	2	3	2	2	3 (8, 12, 13 cycles)
	Dysgeusia	3	4	3	1	
	Fatigue	4	3	1	1	
	Gastrointestinal disorder	3	3	1		1 (9 cycles)
	Alopecia	3	3	1		
	Diabetes	5		1		
	Eye disorder	1		2		1 (9 cycles)
	Infusion reaction	2				
	Thrombosis			1		
	Adrenal insufficiency	1				
	Interstitial pneumonia	1				
	K^+^ decreased	1				

AEs: Adverse events, EV: enfortumab vedotin.

**Table 5:** Timing of onset of AEs.

Adverse Events		CRP-Low Group (*n* = 44)		CRP-High Group (*n* = 17)		*p*-Value in Total Incidence	*p*-Value in Incidence of Grade 3–4 AEs
		No. of Pts, *n* (%)	No. of Grade 3–4 Pts, *n* (%)	No. of Pts, *n* (%)	No. of Grade 3–4 Pts, *n* (%)		
Hematological	Neutropenia	4 (9.1)	3 (6.8)	0 (0)	0 (0)	0.57	0.55
	Anemia	4 (9.1)	1 (2.3)	5 (29.4)	2 (11.8)	0.10	0.19
	Thrombocytopenia	0 (0)	0 (0)	0 (0)	0 (0)	NA	NA
Non-hematological	Skin disorders	31 (63.6)	3 (6.8)	6 (35.3)	0 (0)	0.08	0.55
	Pruritus	19 (43.2)	1 (2.3)	3 (17.7)	0 (0)	0.08	1.00
	Neuropathy	12 (27.2)	0 (0)	0 (0)	0 (0)	<0.05 *	NA
	Dysgeusia	11 (25.0)	0 (0)	0 (0)	0 (0)	<0.05 *	NA
	Fatigue	9 (20.5)	1 (2.3)	0 (0)	0 (0)	0.05	1.00
	Gastrointestinal disorder	5 (11.6)	0 (0)	2 (11.8)	0 (0)	0.68	NA
	Alopecia	6 (13.7)	0 (0)	1 (5.9)	0 (0)	0.66	NA
	Diabetes	3 (6.9)	1 (2.3)	3 (17.7)	2 (11.8)	0.34	0.19
	Eye disorder	4 (9.1)	0 (0)	0 (0)	0 (0)	0.57	NA
	Infusion reaction	2 (4.6)	0 (0)	0 (0)	0 (0)	1.00	NA
	Thrombosis	0 (0)	0 (0)	1 (5.9)	0 (0)	0.28	NA
	Adrenal insufficiency	1 (2.3)	0 (0)	0 (0)	0 (0)	1.00	NA
	Interstitial pneumonia	1 (2.3)	0 (0)	0 (0)	0 (0)	1.00	NA
	K^+^ decreased	0 (0)	0 (0)	1 (5.9)	0 (0)	0.28	NA

AEs: Adverse events, CRP: C-reactive protein, NA: not assessed, Pts: patients * *p* < 0.05 indicates a significant difference.

## Data Availability

Materials and raw data may be given after a request made to the corresponding author.
